# Inhibition of the Glycogen Synthase Kinase 3 Family by the Bikinin Alleviates the Long-Term Effects of Salinity in Barley

**DOI:** 10.3390/ijms231911644

**Published:** 2022-10-01

**Authors:** Jolanta Groszyk, Mateusz Przyborowski

**Affiliations:** Plant Breeding and Acclimatization Institute—National Research Institute, 05-870 Błonie, Poland

**Keywords:** 24-epibrassinolide, brassinosteroids, BR signaling pathway, BZR1, Golden Promise, GSK3, Haruna Nijo, inhibitor, PSII, RNA-Seq

## Abstract

Crops grown under stress conditions show restricted growth and, eventually, reduced yield. Among others, brassinosteroids (BRs) mitigate the effects of stress and improve plant growth. We used two barley cultivars with differing sensitivities to BRs, as determined by the lamina joint inclination test. Barley plants with the 2nd unfolded leaf were sprayed with a diluted series of bikinin, an inhibitor of the Glycogen Synthase Kinase 3 (GSK3) family, which controls the BR signaling pathway. Barley was grown under salt stress conditions up to the start of the 5th leaf growth stage. The phenotypical, molecular, and physiological changes were determined. Our results indicate that the salt tolerance of barley depends on its sensitivity to BRs. We confirmed that barley treatment with bikinin reduced the level of the phosphorylated form of *Hv*BZR1, the activity of which is regulated by GSK3. The use of two barley varieties with different responses to salinity led to the identification of the role of BR signaling in photosynthesis activity. These results suggest that salinity reduces the expression of the genes controlling the BR signaling pathway. Moreover, the results also suggest that the functional analysis of the GSK3 family in stress responses can be a tool for plant breeding in order to improve crops’ resistance to salinity or to other stresses.

## 1. Introduction

Brassinosteroids (BRs) are a class of plant polyhydroxylated steroid hormones, which are involved in many developmental processes and stress responses. Brassinolide (BL), an end-product of the BR biosynthetic pathway, is a signaling molecule recognized by Brassinosteroid Insensitive 1 (BRI1) [1]. The binding of BL by BRI1 leads to conformational changes in this receptor and the disconnection of BRI1 Kinase Inhibitor 1 (BKI1) [2], resulting in its association with BRI1 associated receptor kinase 1 (BAK1) [3,4]. The BRI1-BAK1 transmembrane receptor initiates a cascade of phosphorylation and dephosphorylation of cytoplasmic relay proteins, leading to dephosphorylation and the inactivation of kinases from the Glycogen Synthase Kinase 3 (GSK3) family [5,6,7]. The GSK3 family controls many transcription factors (TFs), e.g., those regulating cell elongation and cell division, root meristem and root development, lateral root development, stomatal development, xylem differentiation, vascular cambial activity, chloroplast development, photomorphogenesis, hypocotyl elongation, floral organ development, flowering, seed development, fruit ripening, sugar content in fruits, and responses to stress (salt, drought, cold, and biotic) [8]. The GSK3s family is represented by four kinase groups [9]; nine are known to date in rice (*Oryza sativa* L.) [10], seven in barley (*Hordeum vulgare* L.) [11], eleven in maize (*Zea mays* L.) [12], and ten in Arabidopsis (*Arabidopsis thaliana* L.) [13]. At low BR concentrations, GSK3 phosphorylates Brassinzaole Resistant 1 (BZR1), one of the major TFs, leading to its proteasomal degradation [14]. The activity of BZR1 is regulated by 14-3-3 protein [15,16] and Protein Phosphatase 2A [17].

The 4-[(5-bromo-2-piridynyl)amino]-4-oxobutanoic acid (bikinin) compound has been identified among the library of 10,000 compounds as one that induces constitutive BR responses in Arabidopsis, i.e., a significant increase in hypocotyl length; long and bending petioles; and blade-shaped, pale-green leaves, as well as effects comparable to those obtained with treatment with BL at micromolar concentrations, in which lateral root density was reduced [18]. Consecutive analyses with bikinin and its inactive variant showed that treatment induced petiole and hypocotyl elongation under light and dark growth conditions, allowing researchers to conclude that Brassinosteroid Insensitive 2 (BIN2–the best characterized kinase from the GSK3 family) is a direct target of this compound. Due to its role in inactivating BIN2, the name of bikinin was derived from its role in the BR signaling pathway, i.e., BIN2 kinase inhibitor. Bikinin folds the lamina joint of barley, similarly to 24-epibrassinolide (24-EBL), and has resulted in a similar phenotype under optimal and salt stress conditions [19].

Previously, BRs have been shown to regulate the fruit ripening of tomato (*Solanum lycopersicon* L.) [20,21], persimmon (*Diospyros kaki* L.) [22], grapevine (*Vitis vinifera* L.) [23], pear (*Pyrus ussuriensis* Maxim.), and apple (*Malus domestica* Borkh.) [24] plants, as well as affecting the yield of crops by regulating grain formation, which has been the best characterized in rice [25,26,27,28,29]. Moreover, BRs have been shown to positively regulate salt stress tolerance in many species, e.g., canola (*Brassica napus* L.) [30], brown mustard (*Brassica juncea* L.) [31], pepper (*Capsicum annuum* L.) [32], pea (*Pisum sativum* L.) [33], tomato [34], potato (*Solanum tuberosum* L.) [35], barley [19], rice [36], and wheat (*Triticum aestivum* L.) [37]. The regulatory role of BRs has been investigated by applying bioactive 24-EBL [38], BL, and 28-homobrassinolide [39] or by using mutants with the genetic dissection of the BR biosynthetic pathway [40,41,42,43,44]. However, the role of GSK3s in barley development using bikinin has not yet been reported. Here we present the results of the application bikinin to barley plants and the long-term effects of GSK3 family inhibition on barley growth under high-salinity conditions. According to the results presented by Honda et al. [44] and Groszyk and Szechyńska-Hebda [45], we considered that the best approach to analyze GSK3s function in barley development was to use a GSK3s inhibitor, i.e., bikinin. We assumed that because of its specificity for GSK3s [46] and its lack of toxic effects, as with another GSK3s inhibitor, i.e., lithium [47,48,49], we would be able to characterize the initial molecular and physiological processes in which GSK3s are involved. The analyses were performed during the outset of the formation of side shoots phase, when tillers were still invisible, i.e., the BBCH20 growth stage according to the BBCH-scale, which is used to identify the phenological development stages of plants [50]. Therefore, the aim of this study was to understand the long-term response of barley plants exposed to salinity to bikinin treatment.

## 2. Results

### 2.1. Bikinin-Induced Phenotypical Responses in Salt-Stressed Barley

**Figure 1:**
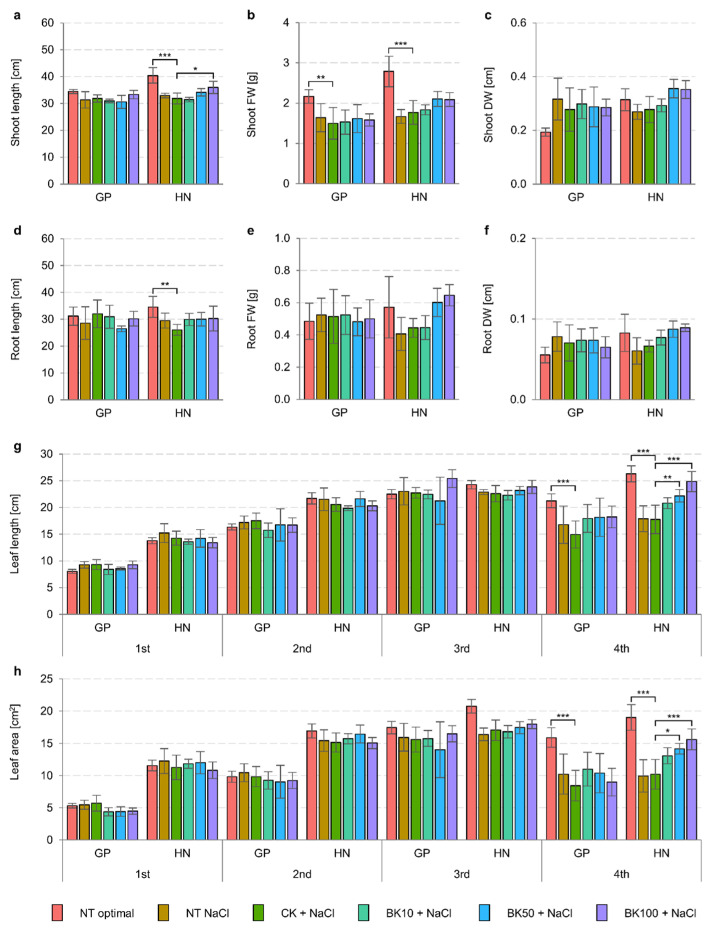
Treatment with bikinin resulted in phenotypic changes in barley grown under normal and salt stress conditions. Shoot length (**a**), fresh (**b**), and dry (**c**) biomass and root length (**d**), fresh (**e**), and dry (**f**) weight, and leaves’ length (**g**) and area (**h**) after bikinin treatment with 10 µM, 50 µM, and 100 µM (BK10, BK50, and BK100, respectively), compared to controls with 0.11% DMSO (CK) used as a background for solvent solutions for each bikinin concentration. Non-treated plants were used as a control for the 0.11% DMSO treatment. The values in (**a**–**h**) represent the mean and standard error for six biological replicates. The results of the HSD Tukey test analysis are presented in Table S1 (*, *p* ≤ 0.05; **, *p* ≤ 0.01; ***, *p* ≤ 0.001).

To test the function of GSK3 in barley development under the effects of salinity, the bikinin treatment was applied to two barley cultivars with differing sensitivities to exogenous BRs, which were previously determined [19]. Golden Promise and more sensitive to BRs Haruna Nijo while growing under control conditions, showed genotype-dependent differences in BBCH20, i.e., shoot length, shoot fresh weight, as well as the areas and lengths of the consecutive leaves (Table 1). Haruna Nijo had 1.2-fold longer shoots, with 1.3-fold higher fresh weight and 1.6-fold higher dry weight compared to Golden Promise under optimal conditions (Figure 1a–c), whereas differences in root parameters were negligible (Figure 1d–f). The most differentiating parameters were the lengths and areas of the consecutive leaves. The 1st, 2nd, 3rd, and 4th leaves of Haruna Nijo were 1.7-fold, 1.3-fold, 1.1-fold, and 1.2-fold longer, with 2.2-fold, 1.7-fold, 1.2-fold, and 1.2-fold greater surface areas, respectively, than those of Golden Promise (Figure 1g,h). Salinity reduced growth parameters in a genotype-dependent manner (Table 1) and barley plants reached the BBCH20 stage ~7 days later. We observed the relevant changes in shoot length and fresh weight and root length. The measured parameters were considerably lower in Haruna Nijo under conditions of salinity (shoot length, 81.6%; fresh weight, 59.6%; dry weight, 85.6%; root length, 85.3%; fresh weight, 71.1%; and dry weight, 73.0%) than in Golden Promise (shoot length, 90.8%; fresh weight, 75.7%; dry weight, 163.9%; root length, 91.6%; fresh weight, 108.2%; and dry weight, 141.0%), indicating the greater resistance of Golden Promise to 150 mM sodium chloride (NaCl). The reduced sensitivity of Golden Promise to salinity resulted in significantly reduced length (79.0%) and area (64.3%) of the 4th leaf, whereas in Haruna Nijo, the reduction in these parameters started from the 3rd and 4th leaf (length, 94.4% and 68.1%; area, 79.0% and 52.3%, respectively). Similarly to previous reports [19], these results indicate the greater sensitivity of Haruna Nijo to salinity.

**Figure 2:**
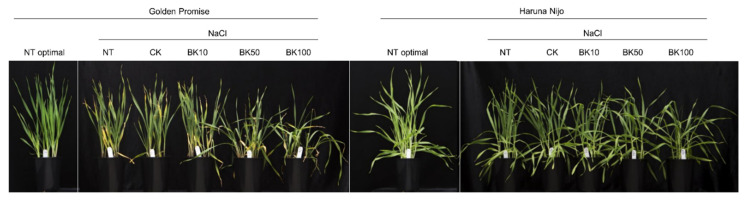
Treatment with bikinin resulted in phenotypic changes in barley growth under normal and salt stress conditions. Barley phenotypes after bikinin treatment with 10 µM, 50 µM, and 100 µM (BK10, BK50, and BK100, respectively) compared to controls with 0.11% DMSO (CK) used as a background for solvent solutions for each bikinin concentration. Non-treated plants (NT) were used as a control for the 0.11% DMSO treatment.

The application of 10 µM bikinin under conditions of salinity increased shoot and root length (112.9% and 114.7%, respectively) and the length and surface area of the 4th leaf (117.2% and 128.2%, respectively). The 50 µM bikinin treatment, applied during salinity, improved growth parameters in Haruna Nijo shoots (length, 107.2%; fresh weight, 118.9%; dry weight, 128.1%; 4th leaf length and area, 124.8% and 139.0%, respectively) and roots (length, 115.0%; fresh weight, 135.7%; dry weight, 131.7%), whereas in Golden Promise the root length (82.5%) and the area of the 1st leaf (76.9%) were reduced. On the other hand, 100 µM bikinin significantly improved the fresh and dry weights of aboveground and belowground organs (shoots, 118.0% and 126.9%; roots, 145.5% and 134.4%, respectively) and the 4th leaf length and area (139.8% and 153.1%, respectively) in Haruna Nijo under the same conditions, suggesting that bikinin at higher concentrations improved barley growth under conditions of salinity. Contrary to this finding, the phenotype of Golden Promise was worse than those of the corresponding controls after the administration of 50 µM and 100 µM bikinin (Figure 2, Table 2).

**Figure 3:**
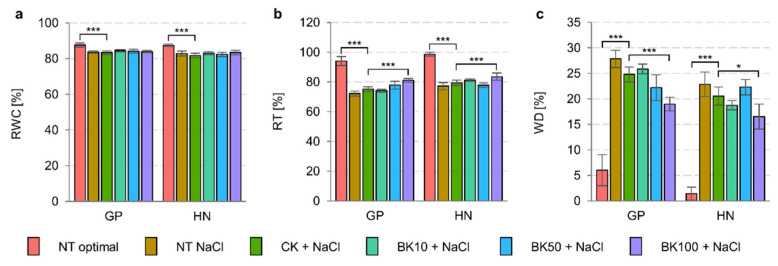
Treatment with bikinin resulted in phenotypic changes in barley grown under normal and salt stress conditions. Relative water content (**a**), relative turgidity (**b**), and water deficit (**c**) after treatment with 10 µM, 50 µM, and 100 µM bikinin (BK10, BK50, and BK100, respectively), compared to controls with 0.11% DMSO (CK), used as background for solvent solutions for each bikinin concentration. Non-treated plants were used as a control for the 0.11% DMSO treatment. The values in (**a**–**c**) represent the mean and standard error for six biological replicates. The results of the HSD Tukey test analysis are presented in Table S2 (*, *p* ≤ 0.05; ***, *p* ≤ 0.001).

Water balance in barley under saline conditions depends on the genotype and the conditions; however, the different genotypes’ reactions to growth conditions were similar (Table 3). Relative water content and relative turgidity, which were reduced under salinity, were improved with bikinin treatment (Figure 3). The water content in the 3rd leaf depended on the barley genotype and the bikinin concentration used (Table 4). In addition, the parameters also depended on the responses of the genotypes to the bikinin treatment, whereas the relative turgidity and water deficit results were significantly different. Water deficit, measured in the 3rd leaf, was increased 4.6-fold in Golden Promise, and 16.2-fold in Haruna Nijo under saline conditions (Figure 3c). The application of 100 µM bikinin significantly reduced water deficits (76.6–80.5%) in both cultivars under saline conditions (Figure 3c).

### 2.2. RNA-Seq Analysis

**Figure 4:**
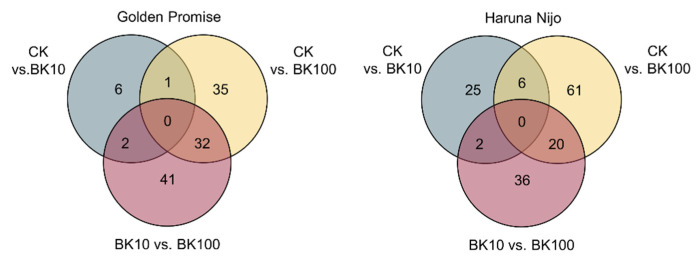
Venn diagrams showing the number of DEGs identified in barley leaves (3rd and 4th) after treatment with two concentrations of bikinin (10 µM and 100 µM) compared with the controls with 0.11% DMSO (CK) used as a background for the solvent solutions for each concentration of bikinin. Common DEGs identified in Golden Promise (**left**) and Haruna Nijo (**right**). DEGs were identified via bioinformatic analysis of three biological replicates of each sample and log2-fold change (<1 and <−1) and Padj < 0.01. Raw data are presented in Table S3.

Based on the phenotypical results, the 3rd and the 4th leaves of barley plants treated with 10 µM and 100 µM bikinin, as well as plants treated with 0.11% DMSO as controls (CK), were used for transcriptome analysis under conditions of salinity. A total of 1235.51 million (1,235,515,176) clean reads were obtained for all samples, with an average of 34.32 million pair-end reads with a size of 100 bp for each sample, and an average of 96.16% Q20. After removing adaptor sequences and low-quality reads, all clean reads were mapped to the barley reference genome IBSC_v2 [51]. The results showed that an average of 29.687 million reads were uniquely mapped to the reference genome (Table S4). To determine the genes regulated by bikinin under saline conditions, the mean normalized counts were calculated, and then used to identify differentially expressed genes (DEGs). Using log2-fold change (<1 and <−1), and Padj < 0.01, we identified 2807 up- and downregulated DEGs (1700 and 1107, respectively) differentiating both barley genotypes under salinity conditions. To capture changes in gene expression during bikinin treatment, we compared the transcript levels of the samples. 117 and 150 genes were identified as a DEGs regulated by 10 µM and 100 µM bikinin in Golden Promise and Haruna Nijo, respectively (Figure 4).

**Figure 5:**
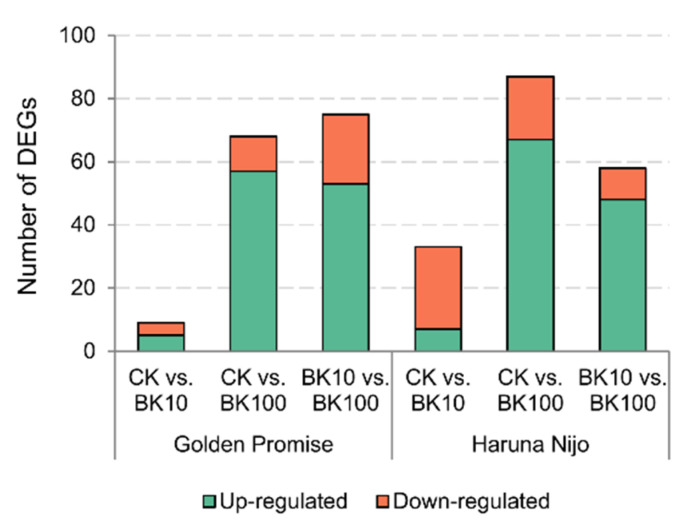
Treatment with bikinin resulted in molecular changes in barley grown under salt stress conditions. Up- and downregulated genes after treatment with 10 µM and 100 µM bikinin (BK10 and BK100, respectively) compared to controls with 0.11% DMSO (CK) used as a background for the solvent solutions used for each bikinin concentration. Non-treated plants were used as a control for the 0.11% DMSO treatment. Results represent the means of 3 biological replicates. Raw data are presented in Table S3.

Between CK and 10 µM bikinin, 5 and 7 genes were upregulated and 4 and 26 were downregulated in Golden Promise and Haruna Nijo, respectively (Figure 5). Then, between CK and 100 µM bikinin, 68 and 87 genes were differentially expressed in Golden Promise (57 upregulated and 11 downregulated) and Haruna Nijo (67 upregulated and 20 downregulated), respectively. A total of 75 DEGs exhibited differential expression patterns between 10 µM and 100 µM bikinin in Golden Promise (53 upregulated, 22 downregulated). In comparison, 48 upregulated and 10 downregulated genes were identified in Haruna Nijo. 12 genes were identified between Golden Promise and Haruna Nijo after bikinin treatment. None of the genes identified after bikinin treatment were found in the group of 2807 DEGs differentiating both cultivars. On the other hand, 15 DEGs were identified as a common bikinin-regulated genes in both genotypes.

**Figure 6:**
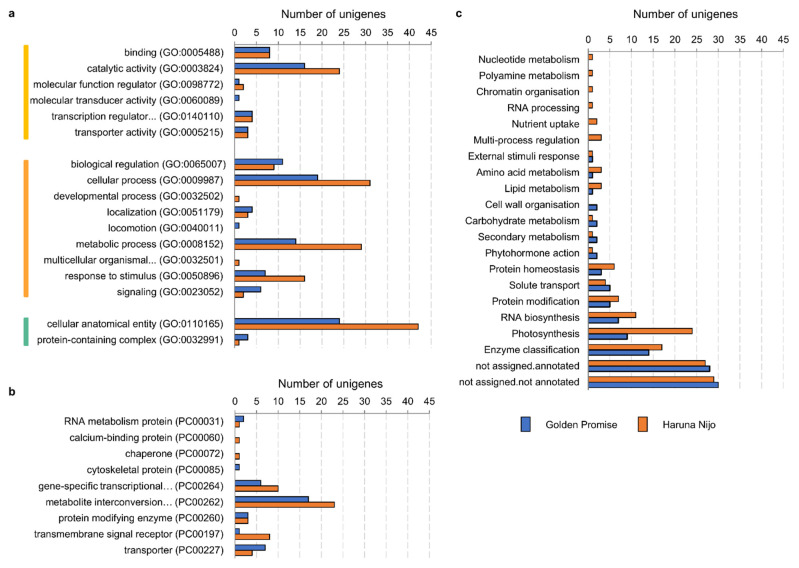
Treatment with bikinin resulted in molecular changes in barley grown under salt stress conditions. Molecular function (yellow), biological process (orange), and cellular component (green) (**a**); protein class (**b**); and gene classification (**c**) after treatment with 10 µM and 100 µM bikinin compared to controls with 0.11% DMSO used as a background for the solvent solutions for each bikinin concentration. Non-treated plants were used as a control for the 0.11% DMSO treatment. Results represent the means for three biological replicates. Raw data are presented in Tables S3 and S4.

According to the Phanter Classification System, a total of 117 genes in Golden Promise and 141 genes in Haruna Nijo were involved in a molecular function, a biological process, and a cellular component (Figure 6a). 15 genes were the same for both barley cultivars. Molecular function included six sub-categories for Golden Promise and five sub-categories for Haruna Nijo, i.e., catalytic activity, binding, transcriptional regulator activity, transporter activity, molecular function regulator, and molecular transducer activity. The biological process contained seven sub-categories for Golden Promise and eight sub-categories for Haruna Nijo, i.e., cellular process, metabolic process, biological regulation, response to stimulus, signaling, localization, developmental process, locomotion, and multicellular organismal process. The cellular component contained two sub-categories for both genotypes, i.e., cellular anatomical entity and protein-containing complex. The protein class contained seven and eight sub-categories for Golden Promise and Haruna Nijo, respectively, among which the metabolite interconversion enzyme, gene-specific transcriptional regulators, and transporter sub-categories possessed the most abundant unigenes (Figure 6b). Moreover, we identified transmembrane signaling receptors, protein modifying enzymes, RNA metabolism proteins, calcium-binding proteins, cytoskeletal proteins, and chaperone protein classes.

A total of 256 genes were annotated in fifteen sub-categories for Golden Promise (112 genes) and twenty one sub-categories for Haruna Nijo (144 genes) (Figure 6c), but 25% and 19% of them were assigned to ‘not assigned.annotated’ and 27% and 20% to ‘not assigned.not annotated’, respectively, suggesting that the functions of more genes were worth exploring. In addition, enzyme classification, RNA biosynthesis, and photosynthesis were the three other main categories, which contained 30 and 52 genes for Golden Promise and Haruna Nijo, respectively. Furthermore, 22 and 27 genes were assigned to protein modification, protein homeostasis, solute transport, carbohydrate metabolism, amino acid metabolism, lipid metabolism, secondary metabolism, phytohormone action, and external stimuli response. Moreover, cell wall organization was characteristic of Golden Promise, and nucleotide metabolism, polyamine metabolism, RNA processing, nutrient uptake, and multi-process regulation were characteristic of Haruna Nijo, with 2 and 9 genes, respectively. Common DEGs for Golden Promise and Haruna Nijo that were regulated as a result of bikinin treatment involved 3 genes representing the group of ‘enzyme classification’, i.e., beta-glucosidase 5, flavonoid 3′monooxygenase CYP75B3, and probably xyloglucan endotransglucosylase/hydrolase protein 28; ‘RNA biosynthesis’, i.e., transcription factor (WRKY); ‘protein modification’, i.e., protein kinase (WAK/WAKL); ‘external stimuli response’, i.e., regulatory protein (CBP60) of systemic acquired resistance; and ‘photosynthesis’, i.e., LHC-related protein (ELIP) and component PsbR of the PS-II complex. Moreover, 3 genes were ‘not assigned’.

### 2.3. Photosystem II Eficiency under Salinity

**Figure 7:**
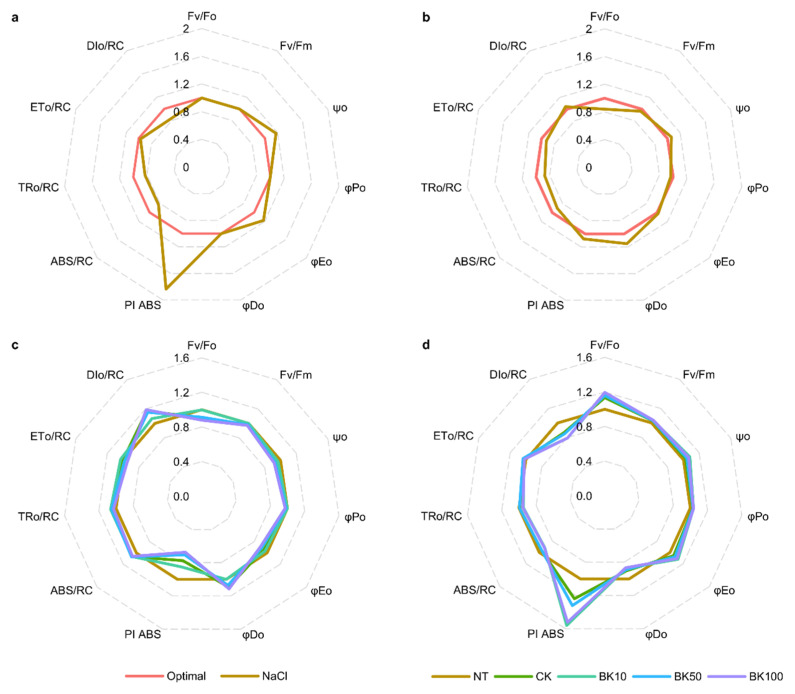
Diagrams presenting photosystem II efficiency under salinity (**a**,**b**) and after bikinin treatment (non-treated; treated with 0.11% DMSO; and treated with 10 µM, 50 µM, and 100 µM bikinin (BK10, BK50, and BK100, respectively) dissolved in 0.11% DMSO (CK)) (**c**,**d**) in Golden Promise (**a**,**c**) and Haruna Nijo (**b**,**d**). Values represent the mean and standard error for 6 biological replicates. The results of HSD Tukey test analysis are shown in Table S4. Fv/Fo, PSII potential activity; Fv/Fm, PSII maximum photochemical efficiency; Ψo, probability (at t = 0) that a trapped excitation moves an electron into the electron transport chain beyond QA; φPo, maximum quantum yield of primary photochemistry (at t = 0); φEo, quantum yield of electron transport (at t = 0); φDo, quantum yield (at t = 0) of energy dissipation; PI ABS, performance index (potential) for energy conservation from an exciton to the reduction of intersystem electron acceptors; ABS/RC, absorption flux (of antenna Chls) per RC; TRo/RC, trapping flux (leading to QA reduction) per RC; ETo/RC, electron transport flux (further than QA−) per RC; DIo/RC, dissipated energy flux per RC (at t = 0). The results of HSD Tukey test analysis are presented in Table S5.

Two-way ANOVA indicated that the efficiency of photosystem II (PSII) was regulated by salinity in both barley cultivars and that the measured parameters depended on genotype, growth conditions, and parameters of quantum yield of the primary PSII photochemistry (Fv/Fo), efficiency and flow of energy (Fv/Fm, ψ0, φP_0_, φE_0_, φD_0_), and performance index (PI ABS) (Table 5). On the other hand, the bikinin treatment results indicated that only electron transport flux per reaction center was genotype-dependent, whereas other measured parameters under conditions of salinity depended on barley’s response to bikinin treatment (Table 6). The analysis of consecutive parameters indicated that under salinity conditions the efficiency of electron transport (φE0, ψ0) was elevated, whereas trapped and dissipated energy flux per reaction center (TR_0_/RC, DI_0_/RC) and absorption flux per reaction center (ABS/RC) were lower in the 3rd leaves of Golden Promise (Figure 7a). Moreover, the performance index (PI ABS) was significantly increased under saline conditions. The opposite effect was observed after bikinin treatment (Figure 7c). The higher the bikinin concentration, the lower the PI ABS and the efficiency of electron transport (φE0, ψ0), whereas the trapped and dissipated energy flux per reaction center (TR_0_/RC, DI_0_/RC) and the absorption flux per reaction center (ABS/RC) increased. Moreover, 50 µM and 100 µM bikinin treatments led to a decrease in the quantum yield of primary PSII photochemistry (Fv/Fo) and an increase in quantum yield for energy dissipation (φDo). A similar but negligible effect was observed with the application of 150 mM NaCl to Haruna Nijo (Figure 7b). Haruna Nijo under saline conditions showed a negligible increased performance index (PI ABS) and reduced quantum yield of the primary PSII photochemistry (Fv/Fo), which resulted in an increased quantum yield for energy dissipation (φDo). The reduced absorption flux per reaction center (ABS/RC) lowered the trapped energy flux per reaction center (TR_0_/RC), consequently lessening electron transport flux per reaction center (ET_0_/RC) and negligibly increasing the dissipated energy flux per reaction center (DI_0_/RC). Bikinin treatment improved the quantum yield of the primary PSII photochemistry (Fv/Fo) and reduced the quantum yield for energy dissipation (φDo) and dissipated energy flux per reaction center (DI_0_/RC) (Figure 7d).

**Figure 8:**
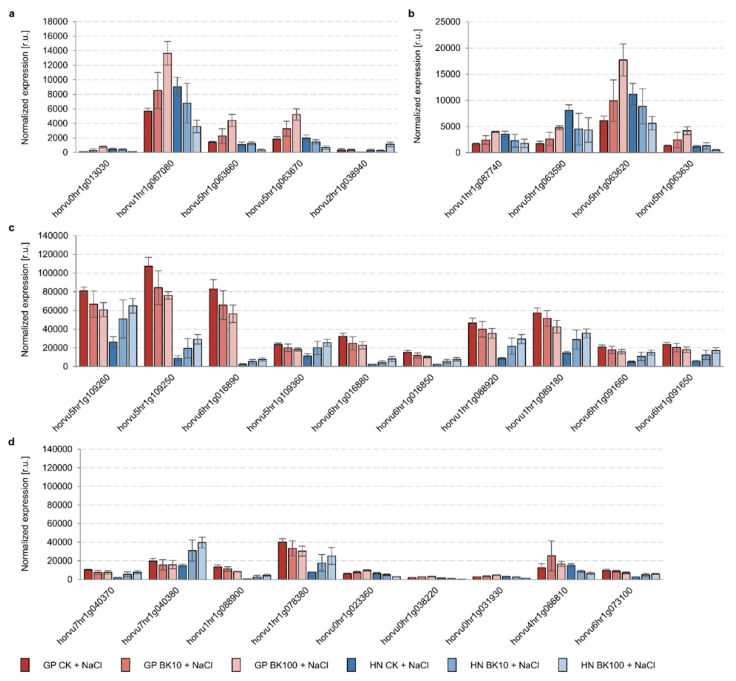
Differentially expressed genes (DEGs) involved in photosynthesis identified in leaves (3rd and 4th) of two barley cultivars, i.e., Golden Promise and Haruna Nijo, after bikinin treatment, identified in both cultivars (**a**), in Golden Promise (**b**), and in Haruna Nijo (**c**,**d**). DEGs were identified via bioinformatic analysis of three biological replicates of each sample and log2-fold change (<1 and <−1) and Padj < 0.01. Identified DEGs encoding (**a**) LHC-related protein (ELIP) (horvu0hr1g013030, horvu1hr1g067080, horvu5hr1g063660, horvu5hr1g063670) and component PsbR of PS-II complex (horvu2hr1g038940); (**b**) LHC-related protein (ELIP) (horvu1hr1g087740, horvu5hr1g063590, horvu5hr1g063620, horvu5hr1g063630); (**c**) component LHCb1/2/3 of the LHC-II complex (horvu5hr1g109260, horvu5hr1g109250, horvu6hr1g016890, horvu5hr1g109360, horvu6hr1g016880, horvu6hr1g016850, horvu1hr1g088920, horvu1hr1g089180, horvu6hr1g091660, horvu6hr1g091650); and (**d**) component LHCb1/2/3 of the LHC-II complex (horvu7hr1g040370, horvu7hr1g040380, horvu1hr1g088900, and horvu1hr1g078380), LHC-related protein (ELIP) (horvu0hr1g023360, horvu0hr1g038220, horvu0hr1g031930, and horvu4hr1g066810), and regulatory factor (CURT) of thylakoid grana stacking (horvu6hr1g073100). Raw data are presented in Table S6.

These results are consistent with the DEGs identified, 5 of which were common for both genotypes (4 genes encoding LHC-related protein (ELIP) and 1 gene encoding the PsbR component of the PSII complex), and 4 other genes encoding ELIP in Golden Promise, with 19 genes encoding component LHCb1/2/3 of the LHC-II complex (14 genes), ELIP (4 genes), and the regulatory factor (CURT) of thylakoid grana stacking (1 gene) (Figure 8). The above results confirmed that the amounts of all DEGs (common characteristics of Golden Promise, and characteristics of Haruna Nijo) in the 3rd and the 4th leaves of barley were opposite, i.e., the amount of genes was increased with a higher concentration of bikinin in Golden Promise. The same concentration of this chemical led to a reduced amount of the genes in Haruna Nijo. All identified DEGs involved in photosynthesis are shown in Figure 8. Moreover, the differing levels of a few genes were compared between Golden Promise and Haruna Nijo under conditions of salinity.

### 2.4. Key Molecular Steps Controlling Consecutive Stages of the BR Signaling Pathway

**Figure 9:**
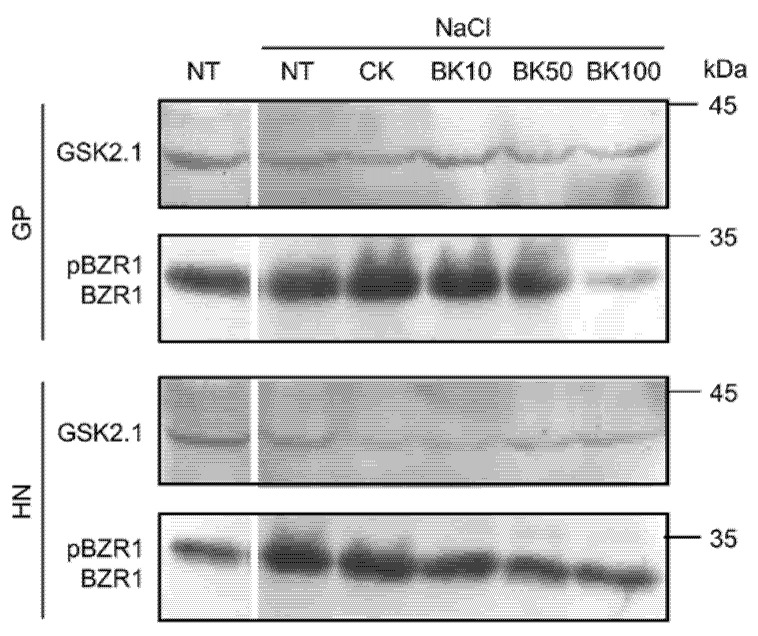
Immunodetection of *Hv*BZR1 and *Hv*GSK2.1. pBZR1 indicates the phosphorylated form of *Hv*BZR1; BZR1 indicates the dephosphorylated form of *Hv*BZR1.

To determinate the contribution of *Hv*BZR1 to the regulation of plant growth under saline stress, the 3rd and the 4th leaf growth of Golden Promise and Haruna Nijo under optimal and salt stress conditions, treated with 10 µM, 50 µM, and 100 µM bikinin, were used for Western blot analysis. Similarly to results obtained in 5-day-old barley shoots [45], in leaves we detected the phosphorylated form of *Hv*BZR1 (Figure 9) and the salinity resulted in an increased accumulation of *Hv*BZR1. The *Hv*GSK2.1 under control and saline conditions were detected in similar amounts. Reduced amounts of *Hv*BZR1 under saline conditions were detected after treatment with 50 µM and 100 µM bikinin in Golden Promise and in all concentrations of the bikinin of Haruna Nijo. In contrast, *Hv*GSK2.1 was detected in the similar quantities.

**Figure 10:**
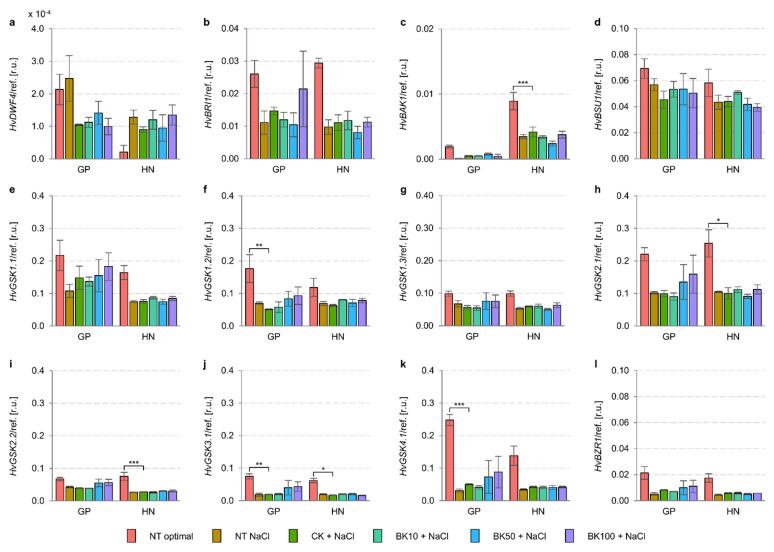
Treatment with bikinin resulted in expression changes in barley grown under normal and salt stress conditions. Expression profile of genes controlling consecutive stages of BR’s biosynthetic (**a**) and signaling pathways (**b**–**l**) after bikinin treatment with 10 µM, 50 µM, and 100 µM (BK10, BK50, and BK100, respectively) compared to controls with 0.11% DMSO (CK) used as a background for solvent solutions for each bikinin concentration. Non-treated plants (NT) were used as a control for the 0.11% DMSO treatment. The values in (**a**–**l**) represent the mean and standard error for three biological replicates. The results of the HSD Tukey test are presented in Table S7 (*, *p* ≤ 0.05; **, *p* ≤ 0.01; ***, *p* ≤ 0.001).

Following the results of RNA-Seq analysis and our observations regarding *Hv*GSK2.1 and *Hv*BZR1 activity, we performed a quantitative analysis of genes controlling the BR signaling pathway (Figure 10). The expression of these genes was similar in Golden Promise and Haruna Nijo under control conditions, except for *HvBAK1*, which was expressed 4.7-fold more highly in Haruna Nijo than in Golden Promise, as well as *HvDWF4* and *HvGSK4.1*, which were expressed 10.2-fold and 1.8-fold more highly in Golden Promise than in Haruna Nijo. These traits may be genotype-dependent, as we have shown previously (Table 7). Salinity led to the inhibition of all the tested genes of the BR signaling pathway in both cultivars, i.e., *HvBRI1* and *HvBAK1* (16.5–42.7%), *HvBSU1* (74.6–82.1%), *HvGSK3s* (12.6–68.4%), and *HvBZR1* (24.1–25.4%). Only *HvDWF4* expression, which controls the first step of the BR biosynthesis pathway, was increased 1.2-fold and 6.1-fold in Golden Promise and Haruna Nijo, respectively. *HvDWF4* expression is regulated by *Hv*BZR1 activity, and the activity of *Hv*BZR1 and the expression of the *HvDWF4* gene have been demonstrated in Arabidopsis, rice, and barley.

After the application of 10 µM, 50 µM, and 100 µM bikinin, there were no significant changes in the expression patterns of the analyzed genes in Golden Promise (Table 8, Figure 10). Only *HvGSK1.1* after the application of 10 µM bikinin and *HvDWF4* after the application of 100 µM bikinin were induced (113.0% and 170.5%, respectively) in Haruna Nijo. However, all concentrations of bikinin induced relevant *HvBRI1* expression (132.9–127.4%) in Haruna Nijo, and only 100 µM bikinin induced the expression of this gene (134.0%) in Golden Promise. Other concentrations of bikinin (10 µM and 50 µM) reduced *HvBRI1* expression (75.6–85.8%) in this barley cultivar. On the other hand, 10 µM bikinin induced *HvBAK1* expression (134.1%) in Golden Promise, whereas 100 µM reduced the expression (83.3%) of this gene in Haruna Nijo. 50 µM of bikinin reduced the expression of all *HvGSK3s* (67.4–88.9%) in Golden Promise, whereas in Haruna Nijo the changes in gene expression were negligible. In contrast, 100 µM bikinin slightly reduced *HvGSK1.1*, *HvGSK1.2*, *HvGSK1.3*, *HvGSK2.1*, *HvGSK2.2*, and *HvGSK3.1* expression (75.8–98.6%) in Haruna Nijo, whereas the expression levels of these genes were negligible in Golden Promise. Only *HvGSK4.1* was reduced (72.1%) after the application of 100 µM bikinin in Golden Promise. Both 50 µM and 100 µM bikinin treatments reduced *HvBZR1* expression (81.2–91.8%) in both barley cultivars under control conditions.

## 3. Discussion

Crops grown under stress conditions show restricted growth and, eventually, reduced yields. Among other treatments, brassinosteroids (BRs) mitigate the effects of stress and improve plant growth. In this study, the following hypotheses were verified: (1) barley’s (*Hordeum vulgare* L.) tolerance of salinity depends on its sensitivity to BRs and the activity of the transcription factor (TF) *Hv*BZR1; (2) treatment of barley with bikinin lowers kinase suppression of *Hv*BZR1, which can be observed through a reduction in the amount of the phosphorylated form of *Hv*BZR1; and (3) the use of two barley varieties with differing responses to salinity allowed the identification of regulatory elements of the BR biosynthesis pathway.

To test our hypotheses, we used two barley cultivars, i.e., Golden Promise and Haruna Nijo, with differing sensitivity to BRs, which was determined by means of the lamina joint inclination test, in which Haruna Nijo presented greater sensitivity [19]. Barley plants with a 2nd unfolded leaf of the same length or ~1 cm longer than the 1st one were sprayed with a diluted series of bikinin, in parallel with the start of NaCl treatment. Barley was grown under controlled conditions until the beginning of the 5th leaf emergence stage. The 3rd and the 4th leaves were used for molecular analyses to determine the effects of the applied inhibitor. Plants treated with 0.11% DMSO were used as a baseline for plants treated with bikinin solutions. Non-treated plants were used as a benchmark for salinity. Similarly to previous studies [19], barley length and fresh weight depended on the genotype’s response to growth conditions, and early leaf length and area were characteristic of the genotype (Table 1). In contrast, the consecutive leaves’ traits depended on the growth conditions (Table 1). Barley’s response to bikinin treatment was genotype-dependent and the greater (albeit insignificant) changes were observed in Haruna Nijo treated with higher concentrations of bikinin (Figure 1 and Figure 2). This was also observed in the growth of the 4th leaf (Figure 1g,h). In contrast to Haruna Nijo, Golden Promise had a faster phenotypic response to bikinin treatment, as observed in the increased length of the 3rd leaf (Figure 1g). These traits may depend on the varieties’ sensitivity to BRs. Despite the greater sensitivity to 24-EBL and bikinin observed in Haruna Nijo, greater changes in lamina inclination were observed at higher concentrations in Golden Promise [19]. BR application promoted hypocotyl and epicotyl elongation of soybeans (*Glycine max* L.) [52], petioles of carrot (*Daucus carota* L.) [53] shoots and roots of barley [19].

Bikinin at the highest concentration also increased the fresh and dry weights of roots in Haruna Nijo under saline conditions, resulting in root lengths similar to plants under optimal conditions (Figure 1). When 5-day-old plants were analyzed, the roots of Haruna Nijo under conditions of salinity were found to be longer than those of Golden Promise [19]. In another study, these changes were associated with the characteristics of root BZR1 transcription factor activity, which has been shown in many studies to be a factor regulating cell division in the Quiescent Center (QC), Columella Cells, and Columella Stem Cell [45,54,55,56]. The QC of the root meristem is controlled by Brassinosteroids at the Vascular and Organizing Center (BRAVO) [57]. The increased cell division levels observed in Haruna Nijo roots after bikinin treatment, resulting in higher fresh and dry weights, may be associated with a higher cell number, but this should be verified in the future. Root phenotype may be connected with the role of Glycogen Synthase Kinase 3 (GSK3) family in the regulation of the auxin signaling pathway [56,58,59] and signal transduction due to the fact that the activity of the *Hv*GSK2.1 kinase from the GSK3 family has not been detected in barley roots [45]. However, *AtSK11* and *AtSK12* have been characterized as a genes that induce root growth under osmotic stress [60], and the best-characterized kinase from GSK3 family, i.e., Brassinosteroid Insensitive 2 (BIN2), has been detected in Arabidopsis roots [61,62]. Orthologs of these genes have been identified in barley [11] and the expression of *HvGSK1.1* in Golden Promise roots was the highest compared to that of other genes from this family [45]. The first functional analysis of *HvGSK1.1* showed that reduced expression by RNAi resulted in higher weights of transgenic plants under conditions of salinity [63]. However, the figure presented in the article shows that the authors of that study compared barley in different developmental stages [63] and that the inhibition of kinase from GSK3 family led to faster growth. Analysis of *OsGSK1* (also known as OsGSK21, OSKζ) with the Os01g10840 locus [10,64,65], an ortholog of BIN2 belonging to a class represented by four orthologs in rice [10] and two in barley [11], showed that knockout mutants had enhanced tolerance to cold, heat, salt, and drought stresses [65]. In contrast, the overexpression of full-length *OsGSK1* led to a stunted growth phenotype, similar to that of the gain-of-function *bin2* mutant [65]. Rice with knock-out of *OsGSK1* exhibited a lower wilting ratio and improved Fv/Fm compared to controls under the same conditions [65]. Similarly, with increasing bikinin concentrations, both barley genotypes had higher relative water content and relative turgidity and lower water deficits in consecutive concentrations than in the controls grown under saline conditions (Figure 3). Despite the changes in both genotypes, the water content was genotype-dependent and depended on the control conditions and the genotypes’ responses to bikinin treatment (Table 3 and Table 4). On the other hand, photosystem II (PSII) activity was genotype-dependent and depended on growth conditions and barley’s response to salinity (Table 5) but changes in consecutive parameters depended on the genotypes’ responses to bikinin treatment (Table 6). Salinity-induced changes were alleviated by bikinin (e.g., PI ABS, DIo/RC in Golden Promise; Fv/Fo, φDo in Haruna Nijo). A positive effect of BR treatment on PSII activity has been observed in cucumber (*Cucumis sativus* L.) [66,67], wheat (*Triticum aestivum* L.) [68,69], soybean (*Glycine max* L.) [70], maize (*Zea mays* L.), spinach (*Spinacia oleracea* L.) [71], and mung beans (*Vigna radiata*) [72]. Differentially expressed gene (DEG) analysis revealed an opposite and genotype-dependent response. 9 out of 117 identified DEGs in Golden Promise and 24 out of 150 DEGs identified in Haruna Nijo played a role in photosynthesis (Figure 8). Previously, the GSK3 family was identified to contain kinases controlling stomata development via the regulation of YDA, MKK4/5, and Speechless [64,73,74,75,76].

The short-term response (30 min or 2 h) to BL or bikinin led to the identification of 272 genes involved in BR metabolism, BR biosynthesis, hormone-mediated signaling, auxin, and the response to abiotic stimuli [18]. The long-term response (approximately 17 days) resulted in the regulation of genes involved in photosynthesis (Figure 7 and Figure 8). The differences indicate that the bikinin response led to rapid changes in phytohormonal regulation that regulated plant phenotype, mainly affecting photosynthetic efficiency and water content in the days following plant growth (Figure 3 and Figure 7).

The most distinctive results were obtained in the response to two concentrations of bikinin. Although the transcript level in Golden Promise increased with higher bikinin concentrations, it decreased in Haruna Nijo and vice versa (Figure 8). However, photosynthetic activity parameters and transcriptome analysis data confirmed the genotype-dependent response to bikinin treatment. According to a previous study [45], we identified a phosphorylated form of *Hv*BZR1 in the 3rd and the 4th barley leaves that accumulated more under conditions of salinity and the amount of which was reduced during treatment with 50 µM and 100 µM bikinin in Golden Promise and after each bikinin concentration in Haruna Nijo (Figure 9). As before, the results indicated two levels of phosphorylation with a lower amount of *Hv*BZR1 over a lower molecular weight. Both proteins, *Hv*BZR1 and *Hv*GSK2.1, detected in leaves showed greater amounts than in 5-day-old barley shoots [45]. In contrast, the expression profiles of these genes showed an opposite response. The low expression profile of *HvBZR1*, accompanied by the high *Hv*BZR1 protein level, suggests that this TF is stabilized through the phosphorylation process and stored in the cytoplasm. To date, many security proteins for BZR1 have been identified [16,77,78,79]. Contrary to the highest expression level of the GSK3 family, protein accumulation in leaves was lower than for *Hv*BZR1. This suggests that *Hv*GSK2.1 may be an unstable protein with short-term activity. Similarly to *HvGSK2.1* and *HvBZR1*, the expression levels of genes controlling the consecutive stages of the BR signaling pathway were reduced by salt stress, but their expression was stable and unchanged after bikinin treatment.

The phenotypic changes observed in the two barley genotypes grown under saline stress confirmed previous results obtained for the same genotypes in a different experimental design. Despite the presence of shared traits, many data suggested a genotype-dependent, exogenous BR-linked response by barley plants to bikinin and the consequent inhibition of GSK3 activities, affecting plant development. Nevertheless, many aspects remain unexplained and further studies are needed to further characterize barley. In summary, our results indicate that barley’s salt tolerance depends on its sensitivity to BRs, but the activity of the *Hv*BZR1 TF should be verified. We confirmed that barley treatment with bikinin reduced the amount of the phosphorylated form of *Hv*BZR1. The use of two barley varieties with different responses to salinity led to the identification of the role of BR signaling in photosynthesic activity. The results suggested that salinity reduces the expression of the genes controlling the BR signaling pathway. Moreover, the results also suggested that the functional analysis of GSK3 in stress responses can be a tool for the breeding of crops to improve plant resistance to salinity or other stresses.

## 4. Materials and Methods

### 4.1. Chemicals

Bikinin (CAS 188011-69-0, purity ≥98%) was purchased from Sigma-Aldrich (Schnelldorf, Germany). Solutions of bikinin (10 µM, 50 µM, and 100 µM) were prepared from 91.5 mM stock, dissolved in 100% dimethyl sulfoxide (DMSO) (Sigma-Aldrich, Schnelldorf, Germany). The controls used in the experiments contained the same concentration of the solvent solution, and these were used as a background for the dilution of bikinin.

### 4.2. Plant Material

The barley (*Hordeum vulgare* L.) cultivars Golden Promise (United States Department of Agriculture, GRAIN-Global, USA, accession number 343079) and Haruna Nijo (Gene Bank Dept., CRI Prague-Ruzyně, accession number 03C0602168) were used in the experiments [45]. Grains were provided from both Gene Bank and imbibed in Petri dishes with three layers of filter paper soaked with spring water (Żywiec-Zdrój S.A., Węgierska Górka, Poland) for 48 h in a refrigerator at 4 °C, then germinated in darkness for 72 h in an incubator at 23 °C, then 8 plants were planted in 12 L buckets (23 cm × 33 cm × 19 cm) filled with solid substrate (Hollas, Pasłęk, Poland) and sand (ratio 4:1), and grown in a greenhouse (October 2019–January 2020) for ~3 months in the case of Haruna Nijo and ~4 months in the case of Golden Promise until complete harvest. Grains from each plant were collected separately and studied as single-seed descent (SSD) lines.

### 4.3. Growth Conditions

SSD lines were used for the experiment. Grains were imbibed and germinated as described above. Then, eight seedlings were planted in 1 L pots filled with soil substrate (Hollas, Pasłęk, Poland) and sand (2:1). Plants were cultivated in a phytotron chamber at a 16 h photoperiod at 20 °C during the day and 18 °C at night, with a daylight intensity of 200 µmol photons m^2^ s^−1^, and a humidity of 70%. Plants were watered to a soil humidity of 70%, with the growth substrate fully watered (as 100%) and fully desiccated (as 0%). At the stage when the 2nd leaf had a similar length to the 1st leaf, sodium chloride application and bikinin treatment were performed. Sodium chloride at a final concentration of 150 mM was administered every second day 3 times with 50 mM NaCl once. Then, pots were watered with tap water to 70% humidity and measured using technical scales and weighed up to 1051 g (fully watered, 1152 g; fully desiccated, 816 g; pot weight, 31 g). Plants watered with tap water only were used as controls and grown under optimal conditions.

### 4.4. Barley Treatment

Barley at the stage described above (1st and 2nd leaf of approximately equal length) was treated with 10 µM (BK10), 50 µM (BK50), or 100 µM bikinin (BK100) or 0.11% DMSO as a control solvent solution (CK). All solutions were prepared as follows:

- BK10: 0.98 µL 91.5 mM bikinin, 8.85 µL 100% DMSO, 25 µL Tween 20 (Sigma-Aldrich, Schnelldorf, Germany), 9 mL deionized water;
- BK50: 4.92 µL 91.5 mM bikinin, 4.91 µL 100% DMSO, 25 µL Tween 20, 9 mL deionized water;
- BK100: 9.83 µL 91.5 mM bikinin, 25 µL Tween 20, 9 mL deionized water; or
- CK (0.11% DMSO): 9.83 µL 100% DMSO, 25 µL Tween 20, 9 mL deionized water.

Barley at the 5th leaf development stage (BBCH20) was used for assessments of molecular and phenotypical characteristics. The experiments were repeated twice.

### 4.5. Physiological Trait Measurements

Barley at the 5th leaf development stage was used for analysis. First, plants were photographed. Second, 5 cm fragments from the 3rd leaf were used for RWC analysis. Third, the 3rd and the 4th leaves were collected and stored at −80 °C for total RNA and protein extractions. Analyses were performed in five and three biological replicates, respectively. In six plants for each growing condition, shoot and root length were measured, and the fresh and dry biomasses of shoots and roots were weighed. Chlorophyll *a* fluorescence was measured using a fluorometer (FluorPen FP 100, ICT International, Australia) and leaf-clips with a window diameter of approximately 3 mm. Chlorophyll *a* parameters were measured according to manufacturer’s protocols with an adaptation to darkness of about 30 min.

### 4.6. Total RNA Extraction, cDNA Synthesis, and Real-Time PCR Analysis

Analyses were performed as described previously. Total RNA was extracted from the 3rd and the 4th leaves using TRI Reagent solutions (Invitrogen, Waltham, MA, USA). Genomic DNA was removed using DNase I, RNase-free (Thermo Fisher Scientific, Waltham, MA, USA). cDNA was synthetized using a Revert Aid cDNA Synthesis Kit (Thermo Fisher Scientific, Waltham, MA, USA). Real-Time PCR was carried out using the 5 x HOT FIREPol EvaGreen qPCR Mix Plus (noROX) (Solis BioDyne, Tartu, Estonia) kit and Rotor Gene 6000q Series (Corbett Life Science, Mortlake, Australia) thermalcycler according to the manufacturer’s protocols. The barley *ADP-rybosilation factor* and *Glyceraldehyde-3-phosphate dehydrogenase* (*GAPDH*) genes were used as the internal controls. For each gene, three biological replicates were performed in three technical repeats, and the average value of the standard curve and standard error was shown. Gene-specific primers used for real-time PCR were published by Groszyk et al. [11] and Groszyk and Szechyńska-Hebda [45].

### 4.7. RNA-Seq Analysis

The mRNA sequencing service was outsourced to the commercial service laboratory of BGI Genomics Co., Ltd. (Hong Kong, China). Analyses were performed using Interdisciplinary Centre for Mathematical and Computational Modelling Warsaw University (Poland) and Galaxy software [80]. Bioinformatics analysis was carried out by filtering out low-quality readings, containing unknown bases (N) and/or low-certainty readings (Q ≤ 20), and removing adapters using trimmomatic v. 0.39 [81]. The next step was mapping the readings to the reference genome of *Hordeum vulgare (*Hordeum_vulgare.IBSC_v2) [82] with the HISAT2 v. 2.2.1 program [83] and calculating the expression levels of the mapped genes using the StringTie v. 2.1.7 program [84]. Expression normalization between the analyzed samples was performed using the DESeq2 tool [85]. For the functional enrichment analysis of DEGs, we used the online Mercator4 v 5.0 tool [86] and the PHANTER 7.0 database [87].

### 4.8. Western Blot Detection of HvGSK2 and HvBZR1

Commercial anti-OsGSK2 (AbP80050-A-SE) and anti-OsBZR1 (AS16 3219) polyclonal antibodies were purchased from Beijing Protein Innovation Co., (Beijing, China) and Agrisera (Vännäs, Sweden), and used to detect *Hv*GSK2.1 and *Hv*BZR1, respectively. Total protein was extracted from the 3rd and the 4th leaves of barley. The plant material was ground to powder in liquid nitrogen and treated with a 1× SDS sample buffer (5 µL per 1 mg ground leaves). Supernatants were denatured at 70 °C for 10 min, centrifuged, and used for SDS-PAGE and immunoblot analysis. Anti-GSK2 and anti-OsBZR1 antibodies were used at dilutions of 1:5000 and 1:10000, respectively. Detection was performed using a PVDF membrane (Bio-Rad Laboratories, Hercules, CA, USA) and AgriseraECL SuperBright, AS16 ECL-S solutions (Agrisera, Vännäs, Sweden).

### 4.9. Data Analysis

Statistical analysis was performed using Microsoft Excel Professional Plus 2016 (Microsoft Office, Warszawa, Poland) and Statistica 13.0 (StatSoft, Kraków, Poland). Graphs were generated using Microsoft Excel Professional Plus 2016 and Microsoft PowerPoint Professional Plus 2016 (Microsoft Office, Warszawa, Poland).

## Figures and Tables

**Table 1:** Results of two-way ANOVA for two barley genotypes (Golden Promise and Haruna Nijo) grown under two conditions (control conditions and salt stress induced by 150 mM sodium chloride) were calculated for aboveground and belowground organ traits (length; FW, fresh weight; DW, dry weight; area). MS—mean square; F—F-test; *p—p-value*; red characters—significant changes.

Organs	Parameters	Genotype			Conditions			Genotype × Conditions		
		MS	F	*p*	MS	F	*p*	MS	F	*p*
Shoot	Length	85.504	18.629	0.000	168.010	36.604	0.000	27.094	5.903	0.025
	FW	0.639	7.950	0.011	4.056	50.464	0.000	0.524	6.519	0.019
	DW	0.00810	3.701	0.069	0.009	4.186	0.054	0.043	19.453	0.000
Root	Length	28.602	1.605	0.220	88.935	4.991	0.037	9.127	0.512	0.482
	FW	0.00146	0.083	0.776	0.023	1.341	0.260	0.063	3.580	0.073
	DW	0.00014	0.456	0.507	0.00000	0.001	0.982	0.003	9.881	0.005
Area of the leaf	1st	255.180	200.257	0.000	1.058	0.831	0.373	0.468	0.367	0.551
	2nd	218.890	133.589	0.000	1.097	0.670	0.423	6.723	4.103	0.056
	3rd	21.015	11.300	0.003	51.744	27.824	0.000	12.152	6.535	0.019
	4th	12.003	2.160	0.157	325.452	58.569	0.000	17.230	3.101	0.094
Length of the leaf	1st	204.050	206.101	0.000	10.415	10.520	0.004	0.088	0.089	0.768
	2nd	141.305	76.308	0.000	0.680	0.367	0.551	1.549	0.836	0.371
	3rd	4.096	2.017	0.171	1.044	0.514	0.482	5.425	2.672	0.118
	4th	56.912	10.419	0.004	247.555	45.321	0.000	23.278	4.261	0.052

**Table 2:** Results of two-way ANOVA for two barley genotypes (Golden Promise and Haruna Nijo) grown under salt stress (150 mM sodium chloride) after bikinin treatment (non-treated; treated with 0.11% DMSO; and treated with 10 µM, 50 µM, and 100 µM bikinin dissolved in 0.11% DMSO) were calculated for aboveground and belowground organ traits (length; FW, fresh weight; DW, dry weight; area). MS—mean square; F—F-test; *p—p*-value; red characters—significant changes.

Organs	Parameters	Genotype			Treatment			Genotype × Treatment		
		MS	F	*p*	MS	F	*p*	MS	F	*p*
Shoot	Length	41.500	12.734	0.001	20.868	6.403	0.000	6.418	1.970	0.114
	FW	1.536	21.918	0.000	0.13566	1.936	0.119	0.112	1.601	0.189
	DW	0.004	1.455	0.233	0.00417	1.486	0.221	0.00753	2.681	0.042
Root	Length	3.651	0.270	0.606	10.016	0.741	0.569	36.755	2.719	0.040
	FW	0.00000	0.000	0.993	0.02550	2.403	0.062	0.04557	4.295	0.005
	DW	0.00024	1.205	0.278	0.00032	1.615	0.185	0.00078	3.931	0.008
Area of the leaf	1st	685.160	435.335	0.000	2.472	1.571	0.197	2.161	1.373	0.257
	2nd	540.606	240.356	0.000	1.051	0.467	0.759	2.831	1.259	0.299
	3rd	36.787	10.374	0.002	3.594	1.013	0.409	3.764	1.062	0.385
	4th	117.695	22.348	0.000	23.623	4.485	0.004	20.049	3.807	0.009
Length of the leaf	1st	402.284	352.902	0.000	2.672	2.344	0.067	1.448	1.270	0.294
	2nd	238.666	93.288	0.000	4.755	1.859	0.132	1.477	0.577	0.680
	3rd	0.003	0.001	0.976	11.367	3.155	0.022	4.618	1.282	0.290
	4th	182.614	29.855	0.000	53.148	8.689	0.000	12.433	2.033	0.104

**Table 3:** Results of two-way ANOVA for two barley genotypes (Golden Promise and Haruna Nijo) grown under two conditions (control conditions and salt stress induced by 150 mM sodium chloride) were calculated for aboveground and belowground organ traits (length; FW, fresh weight; DW, dry weight; area). MS—mean square; F—F-test; *p*—*p*-value; red characters—significant changes.

Parameters	Genotype			Conditions			Genotype × Conditions		
	MS	F	*p*	MS	F	*p*	MS	F	*p*
Relative water content	1.736	1.614	0.219	108.614	100.979	0.000	0.879	0.817	0.377
Relative turgidity	139.466	28.594	0.000	2807.78	575.670	0.000	0.192	0.039	0.845
Water deficit	139.466	28.594	0.000	2807.78	575.670	0.000	0.192	0.039	0.845

**Table 4:** Results of two-way ANOVA for two barley genotypes (Golden Promise and Haruna Nijo) grown under salt stress (150 mM sodium chloride) after bikinin treatment (non-treated; treated with 0.11% DMSO; and treated with 10 µM, 50 µM, and 100 µM bikinin dissolved in 0.11% DMSO) were calculated for the 3rd leaf (length; FW, fresh weight; DW, dry weight; area). MS–mean square; F–F-test; *p–p-value*; red characters–significant changes.

Parameters	Genotype			Treatment			Genotype × Treatment		
	MS	F	*p*	MS	F	*p*	MS	F	*p*
Relative water content	23.872	22.189	0.000	2.890	2.686	0.042	0.664	0.617	0.652
Relative turgidity	210.531	65.462	0.000	89.337	27.778	0.000	22.291	6.931	0.000
Water deficit	210.531	65.462	0.000	89.337	27.778	0.000	22.291	6.931	0.000

**Table 5:** Results of two-way ANOVA for two barley genotypes (Golden Promise and Haruna Nijo) grown under two conditions (control conditions and salt stress induced by 150 mM sodium chloride) were calculated for photosystem II efficiency, measured in the 3rd leaf (Fv/Fo, PSII potential activity; Fv/Fm, PSII maximum photochemical efficiency; Ψo, probability (at t = 0) that a trapped excitation moves an electron into the electron transport chain beyond QA; φPo, maximum quantum yield of primary photochemistry (at t = 0); φEo, quantum yield of electron transport (at t = 0); φDo, quantum yield (at t = 0) of energy dissipation; PI ABS, performance index (potential) for energy conservation from an exciton to the reduction of intersystem electron acceptors; ABS/RC, absorption flux (of antenna Chls) per RC; TRo/RC, trapping flux (leading to QA reduction) per RC; ETo/RC, electron transport flux (further than QA−) per RC; DIo/RC, dissipated energy flux per RC (at t = 0)). MS—mean square; F—F-test; *p*—*p*-value; red characters—significant changes.

Parameters	Genotype			Conditions			Genotype × Conditions		
	MS	F	*p*	MS	F	*p*	MS	F	*p*
Fv/Fo	0.88032	16.604	0.001	0.30702	5.791	0.029	0.30455	5.744	0.029
Fv/Fm	0.00151	15.586	0.001	0.00063	6.458	0.022	0.00061	6.229	0.024
Ψo	0.01086	31.483	0.000	0.03168	91.862	0.000	0.00166	4.802	0.044
φPo	0.00151	15.586	0.001	0.00063	6.458	0.022	0.00061	6.229	0.024
φEo	0.01142	29.127	0.000	0.01659	42.294	0.000	0.00225	5.729	0.029
φDo	0.00151	15.586	0.001	0.00063	6.458	0.022	0.00061	6.229	0.024
PI ABS	10.2159	23.157	0.000	13.7846	31.246	0.000	3.34726	7.587	0.014
ABS/RC	0.08489	11.995	0.003	0.45632	64.479	0.000	0.00012	0.017	0.898
TRo/RC	0.02865	8.559	0.010	0.34348	102.605	0.000	0.00088	0.264	0.614
ETo/RC	0.00150	1.818	0.196	0.01017	12.358	0.003	0.00295	3.588	0.076
DIo/RC	0.01491	15.934	0.001	0.00808	8.637	0.010	0.00169	1.810	0.197

**Table 6:** Results of two-way ANOVA for two barley genotypes (Golden Promise and Haruna Nijo) grown under salt stress (150 mM sodium chloride) after bikinin treatment (non-treated; treated with 0.11% DMSO; and treated with 10 µM, 50 µM, and 100 µM bikinin dissolved in 0.11% DMSO) were calculated for photosystem II efficiency, measured in the 3rd leaf (Fv/Fo, PSII potential activity; Fv/Fm, PSII maximum photochemical efficiency; Ψo, probability (at t = 0) that a trapped excitation moves an electron into the electron transport chain beyond QA; φPo, maximum quantum yield of primary photochemistry (at t = 0); φEo, quantum yield of electron transport (at t = 0); φDo, quantum yield (at t = 0) of energy dissipation; PI ABS, performance index (potential) for energy conservation from an exciton to the reduction of intersystem electron acceptors; ABS/RC, absorption flux (of antenna Chls) per RC; TRo/RC, trapping flux (leading to QA reduction) per RC; ETo/RC, electron transport flux (further than QA−) per RC; DIo/RC, dissipated energy flux per RC (at t = 0)). MS—mean square; F—F-test; *p*—*p*-value; red characters—significant changes.

Parameters	Genotype			Treatment			Genotype × Treatment		
	MS	F	*p*	MS	F	*p*	MS	F	*p*
Fv/Fo	0.05511	0.550	*0.463*	*0.15640*	*1.560*	*0.204*	*0.51010*	*5.088*	*0.002*
Fv/Fm	0.00015	0.768	*0.386*	*0.00029*	*1.512*	*0.217*	*0.00097*	*5.023*	*0.002*
Ψo	0.00143	3.386	*0.073*	*0.00085*	*2.013*	*0.111*	*0.00315*	*7.484*	*0.000*
φPo	0.00015	0.768	*0.386*	*0.00029*	*1.512*	*0.217*	*0.00097*	*5.023*	*0.002*
φEo	0.00054	0.960	*0.333*	*0.00101*	*1.787*	*0.150*	*0.00382*	*6.734*	*0.000*
φDo	0.00015	0.768	*0.386*	*0.00029*	*1.512*	*0.217*	*0.00097*	*5.023*	*0.002*
PI ABS	0.15114	0.230	*0.634*	*0.93804*	*1.426*	*0.243*	*4.23774*	*6.441*	*0.000*
ABS/RC	0.00442	0.751	*0.391*	*0.00455*	*0.773*	*0.549*	*0.02126*	*3.615*	*0.013*
TRo/RC	0.00118	0.492	*0.487*	*0.00230*	*0.956*	*0.442*	*0.00650*	*2.705*	*0.044*
ETo/RC	0.00478	6.349	*0.016*	*0.00175*	*2.326*	*0.073*	*0.00179*	*2.382*	*0.068*
DIo/RC	0.00106	0.866	*0.358*	*0.00133*	*1.089*	*0.375*	*0.00578*	*4.730*	*0.003*

**Table 7:** Results of two-way ANOVA for two barley genotypes (Golden Promise and Haruna Nijo) grown in two conditions (control conditions and salt stress induced by 150 mM sodium chloride) calculated for genes controlling brassinosteroid (BR) biosynthesis (*HvDWF4*) and the BR signaling pathway (*HvBRI1*, *HvBAK1*, *HvBSU1*, *HvGSK1.1*, *HvGSK1.2*, *HvGSK1.3*, *HvGSK2.1*, *HvGSK2.2*, *HvGSK3.1*, *HvGSK4.1*, *HvBZR1*), as determined in leaves (3rd and 4th). MS—mean square; F—F-test; *p*—*p*-value; red characters—significant changes.

Parameters	Genotype			Conditions			Genotype × Conditions		
	MS	F	*p*	MS	F	*p*	MS	F	*p*
*HvDWF4*	7.254 × 10^−8^	12.136	0.008	1.501 × 10^−8^	2.511	0.152	4.055 × 10^−9^	0.678	0.434
*HvBRI1*	2.972 × 10^−6^	0.107	0.752	8.958 × 10^−4^	32.153	0.000	1.687 × 10^−5^	0.605	0.459
*HvBAK1*	6.742 × 10^−5^	34.590	0.001	2.610 × 10^−5^	13.392	0.011	1.114 × 10^−5^	5.715	0.054
*HvBSU1*	4.483 × 10^−4^	2.745	0.136	5.562 × 10^−4^	3.405	0.102	4.232 × 10^−6^	0.026	0.876
*HvGSK1.1*	5.560 × 10^−3^	2.445	0.157	2.954 × 10^−2^	12.987	0.007	3.044 × 10^−4^	0.134	0.724
*HvGSK1.2*	2.580 × 10^−3^	1.288	0.289	1.822 × 10^−2^	9.092	0.017	2.473 × 10^−3^	1.234	0.299
*HvGSK1.3*	1.394 × 10^−4^	0.786	0.401	4.384 × 10^−3^	24.715	0.001	1.508 × 10^−4^	0.850	0.383
*HvGSK2.1*	1.018 × 10^−3^	0.621	0.453	5.366 × 10^−2^	32.746	0.000	6.876 × 10^−4^	0.420	0.535
*HvGSK2.2*	4.380 × 10^−5^	0.298	0.600	4.068 × 10^−3^	27.684	0.001	4.182 × 10^−4^	2.846	0.130
*HvGSK3.1*	1.089 × 10^−4^	1.079	0.329	7.284 × 10^−3^	72.184	0.000	1.467 × 10^−4^	1.454	0.262
*HvGSK4.1*	8.620 × 10^−3^	9.555	0.015	7.711 × 10^−2^	85.485	0.000	9.543 × 10^−3^	10.579	0.012
*HvBZR1*	1.597 × 10^−5^	0.586	0.466	6.408 × 10^−4^	23.531	0.001	7.725 × 10^−6^	0.284	0.609

**Table 8:** Results of two-way ANOVA for two barley genotypes (Golden Promise and Haruna Nijo) grown under salt stress (150 mM sodium chloride) after bikinin treatment (non-treated; treated with 0.11% DMSO; treated with 10 µM, 50 µM, and 100 µM bikinin dissolved in 0.11% DMSO) calculated for genes controlling brassinosteroid (BR) biosynthesis (*HvDWF4*) and the BR signaling pathway (*HvBRI1*, *HvBAK1*, *HvBSU1*, *HvGSK1.1*, *HvGSK1.2*, *HvGSK1.3*, *HvGSK2.1*, *HvGSK2.2*, *HvGSK3.1*, *HvGSK4.1*, *HvBZR1*) as determined in leaves (3rd and 4th). MS—mean square; F—F-test; *p*—*p*-value; red characters—significant changes.

Parameters	Genotype			Treatment			Genotype × Treatment		
	MS	F	*p*	MS	F	*p*	MS	F	*p*
*HvDWF4*	5.578 × 10^−9^	1.695	0.208	7.285 × 10^−9^	2.214	0.104	5.266 × 10^−9^	1.601	0.213
*HvBRI1*	9.469 × 10^−5^	1.643	0.215	4.438 × 10^−5^	0.770	0.557	2.291 × 10^−5^	0.398	0.808
*HvBAK1*	4.489 × 10^−5^	90.158	0.000	3.968 × 10^−7^	0.797	0.547	8.273 × 10^−7^	1.662	0.214
*HvBSU1*	4.866 × 10^−4^	3.593	0.073	6.420 × 10^−5^	0.474	0.754	4.659 × 10^−5^	0.344	0.845
*HvGSK1.1*	3.333 × 10^−2^	17.550	0.000	1.352 × 10^−3^	0.712	0.593	9.785 × 10^−4^	0.515	0.725
*HvGSK1.2*	1.269 × 10^−5^	0.024	0.879	6.822 × 10^−4^	1.287	0.308	3.836 × 10^−4^	0.724	0.586
*HvGSK1.3*	5.851 × 10^−4^	1.451	0.242	1.317 × 10^−4^	0.327	0.857	2.420 × 10^−4^	0.600	0.667
*HvGSK2.1*	1.300 × 10^−3^	0.608	0.445	1.362 × 10^−3^	0.636	0.642	1.411 × 10^−3^	0.659	0.627
*HvGSK2.2*	2.445 × 10^−3^	30.136	0.000	1.713 × 10^−4^	2.111	0.117	7.364 × 10^−5^	0.908	0.478
*HvGSK3.1*	7.127 × 10^−4^	3.072	0.095	2.150 × 10^−4^	0.927	0.468	2.452 × 10^−4^	1.057	0.403
*HvGSK4.1*	2.169 × 10^−3^	1.447	0.243	9.797 × 10^−4^	0.653	0.631	6.860 × 10^−4^	0.457	0.766
*HvBZR1*	6.510 × 10^−5^	4.051	0.058	1.085 × 10^−5^	0.675	0.617	6.768 × 10^−6^	0.421	0.791

## Data Availability

The data presented in this study will be openly available in NCBI SRA at https://www.ncbi.nlm.nih.gov/sra/PRJNA885111 (6 September 2022); reference data, PRJNA885111; temporary submission ID: SUB12107609; release date, 10 October 2023.

## Supplementary Materials

The following supporting information can be downloaded at: https://www.mdpi.com/article/10.3390/ijms231911644/s1.
